# Neuroplasticity in adult second language learning: a systematic scoping review of structural and functional neuroimaging evidence

**DOI:** 10.3389/fnhum.2026.1824559

**Published:** 2026-08-04

**Authors:** Ali H. Alghamdi

**Affiliations:** Faculty of Applied Medical Sciences, Department of Radiological Sciences, University of Tabuk, Tabuk, Saudi Arabia

**Keywords:** brain connectivity, cognitive reserve, functional MRI, neuroplasticity, PRISMA systematic review, second language learning, structural MRI

## Abstract

**Background:**

Adult second language (L2) learning offers a valuable model for studying experience-dependent brain plasticity beyond early development. Neuroimaging studies have reported structural and functional changes associated with L2 acquisition, but findings remain heterogeneous and influenced by differences in study design, learner age, training intensity, and imaging modality. In particular, the relationship between structural remodeling and functional reorganization, and their relevance to aging and cognitive reserve, remains unclear. A narrative synthesis that maps and critically integrates this diverse evidence base is therefore needed to clarify current patterns, limitations, and open questions in adult L2-related neuroplasticity.

**Methods:**

We conducted a systematic scoping review of longitudinal and cross-sectional neuroimaging studies examining adult L2 learning-related plasticity. Following PRISMA 2020 guidelines, literature searches were performed in PubMed, Scopus, Web of Science, and PsycINFO up to June 2025. Fourteen studies on structural MRI, diffusion imaging, functional and MRI methodologies were included. Given substantial heterogeneity in study designs, populations, and outcome measures, findings were synthesized narratively, with particular emphasis placed on longitudinal and intervention-based evidence.

**Results:**

Across studies, adult L2 learning was associated with measurable neuroplastic changes at both structural and functional levels. In younger adults, intensive training was linked to gray matter alterations within frontal and temporal language regions and to white matter reorganization in fronto-temporal pathways. Functional imaging studies reported changes in task-related activation and intrinsic connectivity within language and cognitive control networks. In older adults, evidence for functional reorganization was more consistent than evidence for structural change, while support for L2-related cognitive reserve effects remains limited and preliminary.

**Conclusion:**

The available evidence suggests that adult L2 learning engages dynamic and use-dependent neuroplastic processes, characterized by an interaction between functional reorganization and more gradual structural adaptation. While the adult brain retains a notable capacity for learning-related change, the expression of plasticity appears sensitive to age, training intensity, and methodological factors. Functional reorganization may represent a relatively preserved marker of plasticity in later adulthood. However, claims regarding durable structural change and cognitive reserve require further longitudinal investigation.

**Systematic review registration:**

CRD420251140530.

## Introduction

The human brain is a dynamic organ, capable of significant change throughout life. This fundamental capacity, known as neuroplasticity, underlies the learning of new skills, formation of memories, and adaptation to environmental demands (Zatorre et al., 2012). For decades, the critical period hypothesis posited that the brain’s capability for significant alteration is predominantly restricted to early developmental stages (Lenneberg, 1967). However, an increasing amount of research demonstrates convincing evidence that experience-dependent plasticity continues throughout adulthood, providing a brain foundation for lifelong learning (May, 2011). Among the most complex and ecologically relevant skills that can be learned or further developed in adulthood is a second language (L2). The investigation of adult L2 learning offers a robust framework for examining how the mature brain may alter both its anatomical organization and functional architecture in response to sustained cognitive demands.

Two non-mutually exclusive frameworks help to understand these dynamics. The first is the neural efficiency hypothesis, which states that greater skill proficiency tends to coincide with more efficient neural engagement; this is reflected in reduced or more focal patterns of activity and accompanying structural refinement (Poldrack, 2000). Second, the use-dependent plasticity model predicts the expansion of neural resources during initial learning (Zatorre et al., 2012). Adult L2 learning offers a unique opportunity to examine the transition between these phases.

In this review, the term *second-language learning* is used as a broad construct encompassing structured, explicit, and experience-driven engagement with an additional language during adulthood. By contrast, *second-language acquisition* is used more selectively to describe language development that occurs primarily through naturalistic exposure, immersion, and communicative interaction. This distinction is important because many adult L2 experiences involve a combination of explicit instruction, vocabulary learning, deliberate practice, and acquisition-like processes, whereas first-language acquisition in childhood is predominantly guided by naturalistic developmental mechanisms. Accordingly, unless referring specifically to immersive or naturalistic language exposure, the present review uses the term *L2 learning* as the primary descriptor of the adult language experiences examined in the included studies.

Substantial progress has been made in characterizing the neural correlates of adult L2 learning and language experience, although findings remain heterogeneous across methodologies, populations, and learning contexts. Several key areas are consistently implicated, including the left inferior frontal gyrus (IFG), associated with grammatical processing and lexical retrieval; the superior and middle temporal gyri (STG/MTG), involved in auditory processing and semantic representation; and the anterior cingulate cortex (ACC) and dorsolateral prefrontal cortex (dlPFC), which subserve cognitive control processes essential for language control and selection demands during L2 use (Abutalebi and Green, 2016). Although this cross-sectional literature is invaluable, it primarily depicts the end-state of the language experience in the adult brain; this makes it a challenge to distinguish the effects of learning from pre-existing neural factors that may predispose individuals to successful language acquisition (Rossi et al., 2017). Longitudinal neuroimaging designs are uniquely positioned to support stronger causal inferences about learning-induced plasticity, as they track within-individual brain changes over time and reduce confounding by pre-existing neural differences.

Longitudinal and intervention-based neuroimaging studies have begun to delineate learning-induced structural plasticity during adult L2 learning. For instance, intensive language training in young adults has been associated with measurable increases in gray matter density and cortical thickness across key regions of the core language network (Legault et al., 2019a; Stein et al., 2012) reported that, in native English-speaking Rotary Youth Exchange students learning German during temporary residence in Switzerland, increases in gray matter density in the left IFG and left anterior temporal lobe were associated with improvements in L2 proficiency over an approximately five-month immersive learning period. These findings indicate that the cognitive effort required to acquire a second language can lead to observable changes in pertinent brain regions. However, observed patterns are not consistent; some studies indicate reductions in gray matter, possibly signifying a transition toward more efficient and streamlined neuronal processing. Liu et al. (2021), for example, documented gray matter volume reductions in the left ACC and right IFG over a year of immersive English learning; this correlated with improved language switching ability, hinting at a process of neural optimization. Importantly, longitudinal studies vary substantially in training duration, sampling frequency, control conditions, and outcome metrics, factors that likely contribute to divergent findings across the literature.

In addition to these anatomical findings, research on white matter plasticity indicates that adult L2 learning also influences networks of interregional communication. Using diffusion tensor imaging (DTI), Schlegel et al. (2012) found that learning Chinese over 9 months led to gradual, linear increases in fractional anisotropy (FA)—a marker of white matter integrity—in frontal tracts, which correlated with learning outcomes. Recent extensive research by Wei et al. (2024) expanded on these findings, demonstrating that 6 months of rigorous German language acquisition resulted in enhanced intra-hemispheric connection within a bilateral temporal–parietal network and the right arcuate fasciculus, alongside diminished inter-hemispheric connectivity through the corpus callosum. These alterations in structural connection were directly associated with L2 proficiency, underscoring a significant reconfiguration of the brain’s circuitry to facilitate new linguistic functionality. Although structural plasticity and functional reorganization are often discussed separately, accumulating evidence suggests that they reflect complementary aspects of experience-dependent brain adaptation. Importantly, structural changes may lag behind or constrain functional reorganization, and their temporal coupling may vary depending on training intensity, duration, and the neural systems involved (Fornito et al., 2015; Honey et al., 2009; Taubert et al., 2010).

Functional magnetic resonance imaging (fMRI) studies provide a window into the dynamic shifts in brain activity that underpin behavioral improvements. Effective L2 vocabulary acquisition has been linked to enhanced neural responses. This has been exemplified by diminished activity in the left superior temporal gyrus (LSTG) during tonal discrimination, in addition to the recruitment of a more cohesive and comprehensive network in successful learners, including the supplementary motor area (SMA) and angular gyrus (Yang et al., 2015). The brain’s intrinsic functional architecture is also adaptable beyond task-based activation.

Perhaps one of the most intriguing uses of adult L2 acquisition research is its potential to enhance cognitive and brain health in older adults. The notion of “cognitive reserve” proposes that specific life events can create a buffer against age-associated brain deterioration and cognitive deficits (Stern, 2009). L2 learning engagement has been proposed as a potential contributor to cognitive reserve; however, intervention-based neuroimaging evidence in older adults remains limited, and durable brain-health effects are still uncertain. Intervention studies are now directly testing this hypothesis. For example, Bubbico et al. (2025) found that a four-month English course for older individuals resulted in increased neural activity in the medial prefrontal cortex, which was associated with memory enhancement. However, not all interventions yield positive structural results; the well-powered randomized controlled trial (RCT) by Nilsson et al. (2021) found no substantial alterations in gray or white matter after an 11-week Italian course, despite effective vocabulary acquisition. This may indicate that the nature and intensity of training are essential elements.

Notably, the lack of structural plasticity observed in well-controlled RCTs involving older persons (Nilsson et al., 2021)—despite successful vocabulary acquisition—indicates that age-related limitations on morphological plasticity may dissociate behavioral improvements from neurological alterations. Importantly, only a small subset of studies have directly examined older adults undergoing L2 learning interventions; findings across these studies were heterogeneous, with functional changes observed more consistently than structural alterations. This contrasts with structural effects reported in younger adults undergoing intensive or extended L2 training (Stein et al., 2012; Schlegel et al., 2012; Wei et al., 2024; Hosoda et al., 2013), underscoring age and training intensity as potential limiting requirements. Accordingly, careful attention to study design characteristics, including training intensity, duration, sampling intervals, and the presence of appropriate control groups, is essential for interpreting the strength and generalizability of reported neuroplastic effects.

Despite the recent growth of relevant literature, the field still lacks a comprehensive synthesis integrating evidence across structural MRI, diffusion imaging, and functional MRI in adult L2 learners, spanning younger and older adult cohorts and diverse training paradigms (classroom, immersion, digital). Fundamental questions remain unresolved, and have not yet been adequately addressed by the current evidence base. This includes whether structural and functional changes occur sequentially or independently, how individual differences in age and learning context modulate neuroplastic outcomes, and whether observed neural changes translate into durable cognitive benefits in aging populations.

Although prior reviews have discussed language-related neuroplasticity and bilingual brain adaptation (Abutalebi and Green, 2016; Pliatsikas, 2020), none have systematically mapped multimodal longitudinal and intervention-based neuroimaging evidence focused specifically on adult L2 learning. Importantly, existing syntheses often mix heterogeneous forms of language experience, which can obscure causal inference about learning-induced change. This review addresses these gaps, consolidating existing neuroimaging evidence on structural and functional brain plasticity in adult L2 learning. By critically evaluating and synthesizing longitudinal and cross-sectional studies, we aim to identify consistent patterns of neuroplastic alterations observed across diverse neuroimaging modalities. Meanwhile, we examine how variables such as learning intensity, contextual factors, and learner age influence these neural adaptations. Furthermore, we evaluate current evidence on later-life second-language engagement—including the acquisition of a new language, the strengthening of previously acquired L2 skills, and the continued active use of an existing L2—as a potential contributor to cognitive reserve in older adults. We also critically assess the methodological strengths and limitations of existing studies to identify key directions for future research.

Despite the growing body of neuroimaging literature on adult L2 learning, heterogeneity persists in terms of study design, imaging modalities, training duration, and participant characteristics. In particular, longitudinal evidence is inconsistent, older adult populations are underrepresented, and structural and functional findings are often interpreted in isolation. These limitations complicate efforts to draw coherent conclusions about the nature and timing of learning-induced neuroplasticity and moderating factors. Cross-sectional evidence was included only when it involved adult L2 learners with defined proficiency/exposure and neuroimaging outcomes, and was treated as hypothesis-generating rather than causal. Primary interpretive weight was assigned to longitudinal and intervention-based designs.

Accordingly, the present systematic scoping review integrates longitudinal, intervention-based, and targeted cross-sectional neuroimaging evidence to map structural and functional brain changes associated with adult L2 learning. We critically examine how structural and functional brain changes associated with adult L2 learning vary as a function of age, training intensity, and study design, while explicitly acknowledging current methodological constraints and gaps in the literature.

## Methods

### Protocol and registration

This systematic scoping review was conducted in accordance with PRISMA 2020 guidelines for evidence-mapping reviews (Page et al., 2021). The review protocol was prospectively registered with PROSPERO (CRD420251140530), with *a priori* anticipation of heterogeneity across neuroimaging modalities and outcome measures.

### Eligibility criteria

Studies were selected based on the following pre-defined PICOS (Population, Intervention, Comparator, Outcomes, Study design) framework: (1) Population: the review focused on healthy adults (≥18 years) without a history of neurological or psychiatric disorders, who were engaged in acquiring an L2. Studies involving children, adolescents (<18 years), or clinical populations (e.g., patients with stroke, dementia, aphasia, or developmental language disorders) were excluded. Studies focusing on language expertise populations without clearly defined acquisition phases were excluded to avoid conflating expertise-related neural optimization with acquisition-related plasticity. (2) Intervention/Exposure: The review focused exclusively on adult second language (L2) learning experiences with a clearly defined onset or training period. Eligible exposures included structured classroom instruction, intensive language courses, computerized or digital training programs, study-abroad immersion with a defined learning period, and self-directed learning when accompanied by measurable proficiency development. Studies examining lifelong bilingual status, multilingualism without defined acquisition periods, or professional language expertise (e.g., simultaneous interpreting) were excluded in order to preserve conceptual clarity regarding learning-induced neuroplasticity. Cross-sectional studies were included only when they examined adult L2 learners with defined acquisition histories and reported neuroimaging outcomes relevant to language learning. (3) Comparator: Eligible studies preferentially incorporated a comparator for assessing change or difference, though this was not mandatory for inclusion. This could be a between-groups design (e.g., L2 learners versus a control group of non-learners or monolinguals) or a within-subjects design utilizing pre-training baseline measurements. (4) Outcomes: the primary findings of interest were measures of neuroplasticity derived from brain imaging. Structural metrics included measures of gray matter change (e.g., volume, density, cortical thickness) or white matter integrity (e.g., fractional anisotropy, mean diffusivity from diffusion tensor imaging); functional metrics included changes in brain activity measured by task-based or resting-state functional MRI (e.g., Blood-oxygen-level-dependent (BOLD) signal), or electrophysiological measures from EEG/MEG (e.g., spectral power, event-related potentials, functional connectivity). (5) Study Design: Eligible designs included longitudinal observational studies, randomized controlled trials (RCTs), and pre–post intervention studies examining within-individual brain change during adult L2 learning. Cross-sectional studies were included only when they directly compared adult L2 learners with clearly defined acquisition histories to appropriate control groups, or when they examined associations between L2 proficiency and neuroimaging outcomes. Pure bilingual-versus-monolingual comparisons without defined acquisition trajectories were excluded. Reviews, meta-analyses, case reports, conference abstracts without full results, commentaries, and non-empirical studies were excluded.

### Information sources and search strategy

The literature was systematically searched using main electronic databases: PubMed, Scopus, Web of Science, and PsycINFO. The search strategy was developed to include three fundamental concepts: (“second language learning” OR “L2 acquisition”), (“L2 learning” and “second language training”), (“neuroplasticity” OR “brain plasticity”), and (“neuroimaging” OR “MRI” OR “fMRI” OR “DTI” OR “EEG”). Search strings used a mix of keywords and controlled vocabulary phrases (such as MeSH in PubMed) developed for each database. To ensure comprehensive coverage, the search strategy incorporated multiple synonyms and related terms for L2 learning (e.g., foreign language learning), as well as modality-specific neuroimaging terms (e.g., “structural MRI,” “diffusion MRI,” “resting-state fMRI,” “functional connectivity,” “EEG”). These terms were combined using Boolean operators and adapted as needed for each database.

The search encompassed all publications from 2010 until June 30, 2025. To ensure literature saturation, we also performed backward and forward citation tracking of the reference lists of included studies and relevant review articles.

### Study selection process

**Figure 1:**
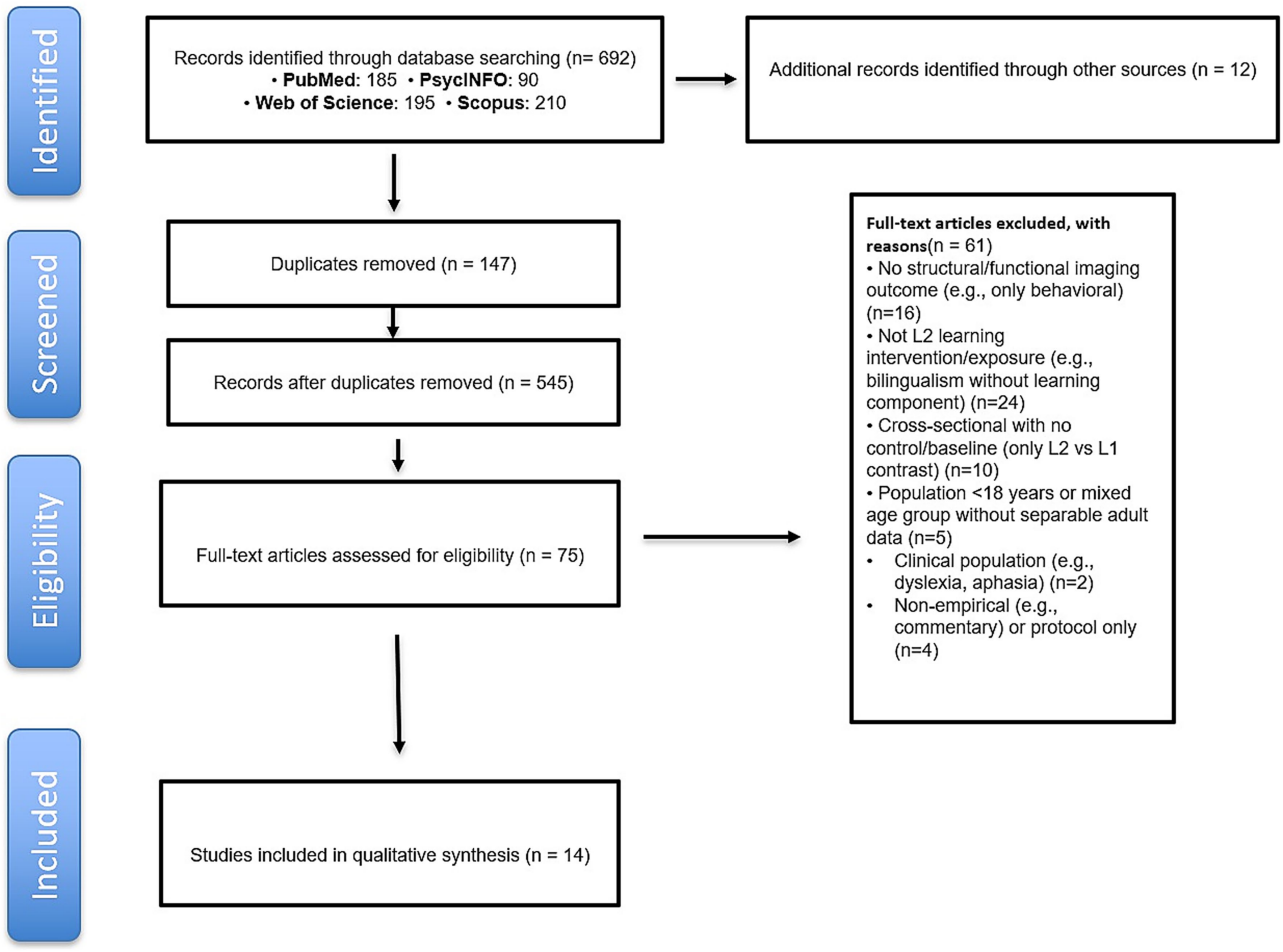
PRISMA 2020 flow diagram illustrating study identification and selection for this systematic scoping review (final included studies, *n* = 14).

Following the search strategy, all identified records were collected, and duplicates were removed automatically and verified manually. The screening process was conducted in two phases:

(1) Title and Abstract Screening: two independent reviewers examined the titles and abstracts of all unique records based on the eligibility criteria. Studies that did not clearly meet the criteria were omitted. (2) Full-Text Assessment: the complete text of each considered paper was obtained and independently assessed by the same two reviewers according to the established PICOS criteria. At both stages, any disagreement between the reviewers was resolved through discussion or, if necessary, by consultation with a third reviewer. The reasons for excluding full-text articles were recorded. The entire selection procedure is detailed in the PRISMA flow diagram (Figure 1).

### Data extraction and management

Data from included studies were systematically extracted using a standardized, piloted data extraction form. Information collected encompassed study identification details such as the first author, publication year, title, and country of the corresponding author. Methodological characteristics were recorded, including imaging modality (e.g., sMRI, fMRI, DTI, EEG), study design (longitudinal, cross-sectional, or RCT), and follow-up duration where applicable. Participant characteristics, such as sample size, age, first and second languages (L1 and L2), and baseline L2 proficiency, were also noted. Details of the intervention or exposure, including the type, duration, and intensity of the L2 learning experience, were documented alongside outcome measures describing specific brain imaging metrics and regions investigated. Quantitative results, including effect sizes (e.g., Cohen’s *d*, *η*^2^), correlation coefficients (*r*), *p*-values, and confidence intervals, were extracted where available, along with any relevant methodological notes or limitations reported by the original authors. When neuroimaging outcomes were missing, incompletely reported, or inconsistently defined across studies, data were extracted at the highest level of detail available and synthesized narratively; no studies were excluded solely on the basis of incomplete reporting, unless core methodological information required for eligibility assessment was unavailable. Data extraction was independently conducted by two reviewers, and any discrepancies were resolved through discussion and consensus to ensure accuracy and reliability.

### Data synthesis (systematic scoping approach)

Given the substantial heterogeneity across included studies—including variability in learner age, L2 exposure, training intensity, neuroimaging modality, outcome metrics, and analytical frameworks—a quantitative meta-analysis was not appropriate. Instead, a systematic scoping synthesis was conducted to map the extent, nature, and consistency of neuroimaging evidence on adult L2 learning-induced plasticity.

Studies were grouped by imaging domain (structural MRI, diffusion imaging, task-based functional imaging, and resting-state or electrophysiological measures) and critically compared with respect to directionality of effects, learner characteristics, and methodological quality. Consistent with the objectives of a scoping review, all eligible studies contributed equally to the narrative synthesis following application of the predefined inclusion and exclusion criteria. This approach enabled integrative evaluation of convergent patterns, boundary conditions, and unresolved discrepancies across the literature.

Consistent with scoping review methodology, the aim was to characterize evidence patterns and gaps rather than to estimate pooled effect sizes. This synthesis approach was specified *a priori* due to anticipated heterogeneity across imaging modalities, study designs, and outcome metrics; it therefore does not represent a *post hoc* deviation from the registered protocol.

## Results

Consistent with a scoping review framework, the results emphasize mapping patterns, consistencies, and sources of divergence across studies rather than quantitative aggregation of effect sizes.

### PRISMA flow and study selection

A thorough literature review was performed across four principal databases—PubMed, Scopus, Web of Science, and PsycINFO—to find relevant neuroimaging studies investigating structural and functional brain plasticity associated with adult L2 learning. The search was completed on June 30, 2025, and yielded a total of 692 records, including 185 from PubMed, 210 from Scopus, 195 from Web of Science, 90 from PsycINFO, and 12 additional records identified through other sources, such as reference tracking and citation alerts.

After removing 147 duplicates, 545 unique records were evaluated according to titles and abstracts. Out of these, 470 studies were removed for not meeting the inclusion criteria, primarily owing to irrelevance to L2 neuroimaging or lack of an intervention component. The remaining 75 full-text articles were retrieved and assessed for eligibility according to the predefined PICOS framework.

Following full-text evaluation under the revised eligibility criteria, a total of 61 articles were excluded for the absence of neuroimaging outcomes (*n* = 16; e.g., studies limited to behavioral data); a lack of focus on L2 learning interventions or exposures (*n* = 24; e.g., bilingualism-only comparisons and professional interpreting expertise studies); cross-sectional designs without control or baseline measures (*n* = 10); non-adult or mixed-age samples without separable adult data (*n* = 5); involvement of clinical populations, such as dyslexia or aphasia (*n* = 2); and as non-empirical publications, including commentaries or protocol papers without results (*n* = 4).

Finally, 14 studies met all inclusion criteria and were incorporated into the qualitative synthesis. These studies represent the currently available neuroimaging evidence examining structural and functional brain plasticity during adult second language learning with clearly defined acquisition periods. The included studies employed structural MRI, diffusion imaging, and functional MRI (task-based and resting-state) methodologies across diverse learning contexts, including classroom-based instruction, intensive training programs, immersion experiences, and digital language learning platforms.

The comprehensive selection process is depicted in the PRISMA 2020 flow diagram (Figure 1), which records the quantity of studies identified, screened, excluded, and included at each stage of the review. This transparent reporting ensures full traceability of how studies progressed from initial identification to final inclusion in the synthesis.

### Descriptive summary of included studies

The included studies, detailed in Table 1, were published from 2012 to 2025, with a notable rise in publications occurring from 2019. Geographically, the research was conducted across many regions, including Europe, North America, and Asia. The predominant neuroimaging modality was structural MRI, used in the majority of studies, followed by diffusion imaging examining white matter microstructure and connectivity. Functional MRI studies included both task-based and resting-state designs. No EEG-only studies met the revised inclusion criteria. This distribution reflects the field’s primary reliance on structural and MRI-based functional methods for investigating adult L2-related neuroplasticity. The predominant neuroimaging modality was structural MRI, used in 7 of the 14 included studies (50%). Diffusion imaging techniques examining white matter microstructure and connectivity were employed in 5 studies (36%). Functional MRI was used in 4 studies (29%), including 2 task-based designs (14%) and 2 resting-state investigations (14%). Several studies employed multimodal imaging approaches, resulting in overlapping modality representation across categories.

**Table 1:** Study characteristics.

Study (Year)	Country	Design	Population (*n*)	Age and gender distribution	Modality	Intervention/exposure	Primary outcome(s)
Yang et al. (2015)	USA/China	Longitudinal controlled	39 adults (learners)	23 participants, 12 females; mean age = 20.61 years, SD = 1.04, range = 18.58–23.33 years	fMRI	6-week Chinese pseudoword training	BOLD activation, effective connectivity
Liu et al. (2021)	China	Longitudinal observational	20 English majors	mean age: 18.43 years, SD: 0.59, 18 participants were females	sMRI (VBM)	1-year academic immersion	Gray matter volume, language switch cost
Legault et al. (2019a, 2019b)	USA	Longitudinal with control	24 learners, 20 controls	Learners: mean 20.58 (SD not reported) Controls: mean 21.95 (SD 1.35)	sMRI	4-month university Spanish course Classroom-based intermediate Spanish learning (lexical decision, semantic judgment tasks); controls had no L2 exposure	CT increases in left ACC and right MTG; GMV increases in left IFG (higher SSJ accuracy) and right IFG (earlier AoA); CT in right MTG correlated with English–Spanish discrimination ability; ACC CT increase correlated with ACC–MTG functional connectivity; no CT/GMV change in controls
Stein et al. (2012)	Switzerland	Longitudinal observational	10 exchange students	(3 males, 7 females) Mean age 17.5 years (range 16–18.5)	sMRI (VBM)	~5-month immersive German learning Naturalistic German L2 learning during exchange; 3-week intensive course before baseline; 5 months immersion; proficiency measured by cloze + vocabulary tests	↑ GM density in left IFG and left ATL correlated with Δ L2 proficiency; lIFG reflects learning-specific structural plasticity; lATL partially influenced by immersion/maturation; absolute GM density not correlated with absolute proficiency; structural changes track individual learning gains, not prior skill
Wei et al. (2024)	Germany	Longitudinal cohort	84 Arabic speakers	84 baseline; 59 at 3 months; 51 at 6 months Majority male (51/59 at 3 months; 43/51 at 6 months) Mean 24.5 y	DTI	6-month intensive German course	Late-phase (3–6 mo) increases in bilateral temporal–parietal and right AF connectivity; significant decreases in interhemispheric CC connectivity; connectivity changes correlated with L2 proficiency; right-hemisphere subnetworks critical for vocabulary and lexical-semantic learning
Legault et al. (2019a, 2019b)	USA/Taiwan	RCT	36 trainees, 15 controls	Picture–Word association (PW) group: 17 3D Virtual Environment learning (VE) group: 19 L2 groups: mean 21.9 y; Controls: 20.47 y	sMRI	7-session Mandarin vocabulary training; PW: 2D picture–word; VE: 3D virtual environment; 90 nouns	Both groups: CT ↑ in bilateral IFG, left ACC, IPL/SMG, STG, right MFG/SPL; GMV ↑ in left putamen. VE group: greater right ACC GMV; right IPL/SMG CT correlated with learning. PW group: right IFG CT correlated with learning. Structural plasticity detectable within ~20 days; learning context shapes neural substrates engaged.
Rossi et al. (2017)	USA	cross-sectional	25 L2 learners, 24 monolinguals	18–27 years (range; mean not reported)	DTI	Native English speakers, late L2 Spanish; average L2 AoA ≈ 12.1 years; mean immersion ≈ 6.3 months; intermediate L2 proficiency (self-rating ≈ 7.2/10; naming accuracy ≈ 0.55; DELE composite ≈ 0.53)	L2 learners showed higher FA than monolinguals in left-dominant white-matter network (ATR, IFOF, UF, ILF, corona radiata, internal capsule, posterior thalamic radiation); no regions where monolinguals > L2. Mean FA in these tracts correlated negatively with AoA (earlier AoA → higher FA), but showed no significant correlation with L2 proficiency or length of immersion.
Hosoda et al. (2013)	Japan	(1) Cross-sectional MRI; (2) 16-week longitudinal training	Cross-sectional: 137 native Japanese (71F), right-handed; Cohort: TG = 24 (14F), CG = 20 (10F)	Cross-sec: M = 24 (18–42); Cohort: M = 20	multi-modal MRI	16-week L2 vocabulary program (~1,000 words)	Cross-sectional: EVT correlated with GM in IFGop, caudate, STG/SMG (right-dominant) and FA in right IFG/ILF/AF; Right IFGop–caudate and right IFGop–STG/SMG connectivity correlated with EVT. Longitudinal: TG showed GM increase in right IFGop, FA increase under right IFGop, and increased right IFGop–caudate and dorsal-pathway connectivity; all correlated with TOEIC gains. Changes reversed at 1-year unless L2 practice continued.
Schlegel et al. (2012)	USA	Longitudinal cohort	27 adults (11 learners, 16 controls);	15F; Mean 20.05 (SD 1.89)	DTI	Intensive Modern Standard Chinese course (9 months; 9 classes/week)	Learners showed progressive FA increases and decreases in left language tracts, right temporal regions, and prominently in frontal tracts crossing the genu of the corpus callosum. 16 tracts showed significant FA increases (5 projecting to caudate); no FA decreases. FA slope correlated with learning success.
Bubbico et al. (2019)	Italy	RCT	27 older adults (FLL = 14; Control = 13)	59–79 years old	rs-fMRI	16-week beginner English course (weekly 2 h classes, homework, communicative tasks; control received no training)	FLL increased bilateral fALFF in dmPFC and OFC (anterior DMN). mPFC activity increases correlated with gains in semantic long-term memory. FLL improved MMSE and delayed verbal memory; controls showed no neural changes.
Nilsson et al. (2021)	Sweden	RCT	160 older adults (65–75 yrs) randomized; MR subsample = 72 (Language *n* = 38; Relaxation *n* = 34)	65–75 years (Mean ~69.3 ± 2.7 language; 69.9 ± 2.9 relaxation)	multi-modal MRI	11-week entry-level Italian course (55 h teacher-led + homework) vs. Relaxation training (active control)	No structural brain changes in IFG, STG, hippocampus, or WM tracts (SLF, ILF, IFO, cingulum). No gray/white matter changes correlated with vocabulary learning. Baseline hippocampal volume and associative memory ability predicted vocabulary proficiency.
Fong et al. (2022)	Hong Kong	Longitudinal observational	20 older adults	58–69 years (Mean = 63.7; SD = 2.9)	sMRI	Two-month intensive Italian vocabulary learning program: 21 visits total; 10 computerized 2-h vocabulary lessons (learning 60 verbs + 360 nouns), 4 revision sessions, cognitive and phonological battery, and final test; introductory lesson before MRI	Predictors of vocabulary learning: Structural measures of left pars orbitalis and left caudal middle frontal cortex predicted both immediate and long-term learning success. Entorhinal cortex, insula, and other cortical regions also contributed. Thalamic volume predicted final retention. Cognitive predictors included semantic fluency, verbal episodic memory (HKLLT), picture naming, processing speed, and phonological awareness. Findings support central roles of semantic and episodic memory in older-adult vocabulary learning.
Bubbico et al. (2025)	Italy	RCT	27 older adults (FLL: *n* = 14; Control: *n* = 13)	Older adults; exact mean age not provided but all were ≥older-adult range	rs-fMRI	Intervention: 16-week beginner-level English course; weekly 2-h sessions (90-min class + 30-min homework). Curriculum covered vocabulary, grammar, communication, and cultural aspects. Control: No training; monthly check-ins to maintain usual lifestyle	FLL increased resting-state activity in the medial prefrontal cortex (mPFC) including dorsomedial PFC and orbitofrontal cortex (DMN–anterior portion). Significant correlation between increased mPFC fALFF and improved semantic long-term memory (Babcock Memory Recall Test). No significant changes in controls. Findings interpreted as compensatory neural plasticity in aging.
Schultz et al. (2024)	USA	Pre–post longitudinal intervention study (no control group)	41 healthy older monolingual adults	60–80 years (M = 66.63, SD = 4.7)	fMRI	4-month online L2 course (Rosetta Stone); participants chose Spanish/French/German/Italian/Japanese; 90 min/day, 5 days/week	Increased incongruent > congruent activation in PFC, parietal cortex, ACC; degree of neural change positively correlated with total time spent learning the L2. Suggests enhanced executive control and functional neuroplasticity.

This table provides a comprehensive overview of the 14 studies included in our synthesis, detailing their geographical origin, design, participant profiles, imaging modalities, and the nature of the second language learning intervention. Studies involving professional simultaneous interpreters are included as boundary cases of long-term language control expertise and are not interpreted as evidence of second language acquisition or learning-induced plasticity.

Study designs in the revised evidence base were predominantly longitudinal and intervention-based. Thirteen of the fourteen included studies (93%) employed longitudinal, pre–post, cohort, or randomized controlled designs examining within-individual brain change during adult L2 learning. Only one study (7%) used a purely cross-sectional design. This distribution reflects the stricter focus on learning-induced neuroplasticity and substantially strengthens the interpretive weight of within-individual change over time relative to cross-sectional association. Randomized controlled trials constituted four of the 14 studies (29%), further enhancing the internal validity of the evidence base (Bubbico et al., 2025; Nilsson et al., 2021; Bubbico et al., 2019; Legault et al., 2019b). Participant populations included both younger and older adults engaged in structured second-language learning interventions. Younger-adult cohorts were more frequently exposed to intensive training programs, immersion experiences, or university-based language courses, whereas older-adult cohorts typically participated in structured classroom-based, home-based, or digitally supported language-learning interventions designed to examine learning-related neuroplasticity and potential cognitive benefits.

Across studies, training intensity varied substantially and was operationalized inconsistently. For the purposes of this review, intensive training was defined descriptively as structured language-learning programs involving daily or near-daily engagement over several weeks or months, irrespective of participant age. However, interventions meeting this criterion were more commonly represented in younger-adult cohorts, whereas older-adult studies generally involved lower-intensity but still structured language-learning programs.

### Synthesis of structural plasticity findings

#### Gray matter changes

Several longitudinal and intervention-based studies reported experience-dependent changes in gray matter structure following adult L2 learning. Specifically, increases in gray matter density in the left inferior frontal gyrus and anterior temporal regions have been associated with gains in L2 proficiency (Stein et al., 2012). In addition, training-related changes in cortical thickness and gray matter volume within frontal and temporal language regions have been reported following short-term vocabulary learning (Legault et al., 2019a, 2019b).

Across studies, increases in cortical thickness or gray matter volume were observed in language-related neocortical regions including the IFG, MFG, STG, anterior cingulate cortex, and hippocampus, following periods of intense L2 learning (Stein et al., 2012; Legault et al., 2019a, 2019b). For instance, Liu et al. (2021) observed a decrease in gray matter volume in the left ACC and right IFG over a year of immersion learning, interpreted as possible reflections of enhanced neuronal efficiency. In addition, Nilsson et al. (2021) conducted an RCT in older adults and found no significant gray matter changes following an 11-week language course. Limited available evidence raises the possibility of age-related differences in structural plasticity; however, current findings are based on a small number of heterogeneous studies and should be interpreted cautiously.

#### White matter changes

Longitudinal diffusion imaging studies provide evidence that adult L2 learning can induce measurable alterations in white matter organization and connectivity. However, the extent and specific conditions under which these changes occur differed across study designs and training paradigms. Schlegel et al. (2012) reported a linear rise in FA in frontal tracts throughout a nine-month period of Chinese language learning, which was associated with educational results. Wei et al. (2024) reported dynamic reorganization of white matter networks during 6 months of intensive German learning, characterized by increased intra-hemispheric connectivity and decreased inter-hemispheric connectivity via the corpus callosum.

Notably, apparent inconsistencies across studies were closely linked to differences in age group, training intensity, and study design, suggesting that structural plasticity is highly contingent rather than uniform.

### Synthesis of functional plasticity findings

#### Task-based activation and connectivity

Task-based fMRI studies indicate that adult L2 learning is associated with functional reorganization in networks supporting language processing and cognitive control. Yang et al. (2015) reported that proficient learners exhibited enhanced activity in the left SMA, STG, and right angular gyrus during tone identification tasks. In older people, Schultz et al. (2024) observed reduced activation in the left dlPFC during a Stroop task following a 12-week L2 learning intervention; this corresponded with enhanced behavioral performance. Compared with structural findings, functional reorganization was more consistently observable across age groups, supporting its role as a potentially more sensitive marker of adult learning-related plasticity.

#### Resting-state functional connectivity

Changes in intrinsic brain networks were also evident. In an older cohort, Bubbico et al. (2025, 2019) demonstrated that L2 learning increased resting-state functional connectivity and fractional amplitude of low-frequency fluctuations in prefrontal regions, which correlated with improvements in global cognition and memory.

#### Directional summary of neuroplastic effects

Reported findings most frequently described increases in neuroplastic metrics such as cortical thickness, activation, and connectivity. Nevertheless, notable instances of decreased metrics were reported, which were frequently interpreted as neural refinement or improved efficiency (Liu et al., 2021).

### Identification of data gaps and reporting issues

During data extraction, various discrepancies and difficulties were observed, as detailed in Table 2. The absence of a control group was the most prevalent issue, observed in numerous longitudinal and pre–post studies, restricting the possibility of specifically attributing changes to L2 acquisition. Other frequent issues included small sample sizes, missing statistical values (e.g., exact *p*-values or effect sizes), and unclear quality assessment ratings. Furthermore, although the revised evidence base is predominantly longitudinal, some cross-sectional designs remain limited in their ability to disentangle pre-existing neural differences from learning-induced plasticity.

**Table 2:** Key results across studies.

Study (year)	Plasticity domain	Main finding	Direction of effect
Yang et al. (2015)	Functional activation/connectivity	Successful learners showed greater activation in SMA, STG, Angular Gyrus; more coherent network connectivity.	↑
Liu et al. (2021)	Structural GM	GMV decrease in ACC and IFG correlated with reduced language switch cost.	↓ (efficiency)
Legault et al. (2019a, 2019b)	Structural GM	Increased cortical thickness in right MTG and left ACC.	↑
Stein et al. (2012)	Structural GM	GM density increases in left IFG and ATL correlated with proficiency gain.	↑
Wei et al. (2024)	Structural WM	Increased intra-hemispheric connectivity, decreased interhemispheric connectivity.	↑/↓
Legault et al. (2019a, 2019b)	Structural GM	Increased CT in IFG, ACC, STG, IPL; increased GMV in putamen.	↑
Rossi et al. (2017)	Structural WM	Higher FA in multiple tracts in L2 learners vs. monolinguals.	↑
Hosoda et al. (2013)	Structural GM/WM	GMV and FA increase in right IFG; changes correlated with proficiency.	↑
Schlegel et al. (2012)	Structural WM	FA linearly increased in frontal tracts; correlated with learning outcome.	↑
Bubbico et al. (2019)	Functional connectivity	Increased rs-FC in rIFG, rSFG, SPL; correlated with MMSE improvement.	↑
Nilsson et al. (2021)	Structural GM/WM	No significant GM or WM changes post-intervention.	Null
Fong et al. (2022)	Structural GM	Pre-learning cortical curvature predicted learning success.	N/A (predictor)
Bubbico et al. (2025)	Functional connectivity	fALFF increase in mPFC correlated with memory improvement.	↑
Schultz et al. (2024)	Functional activation	Reduced dlPFC activation during Stroop task post-learning.	↓ (efficiency)

Findings from simultaneous interpreter studies reflect neural adaptations associated with prolonged professional expertise and are not interpreted as evidence of second language learning or acquisition. Population-based bilingualism studies are included to contextualize cognitive reserve and aging-related neural variability and are not interpreted as evidence of second language learning or acquisition.

## Discussion

The current scoping review synthesized longitudinal and cross-sectional neuroimaging evidence from 14 studies to map the trajectory of structural and functional brain plasticity in healthy adults acquiring a second language. The findings of this review can be most coherently interpreted through two complementary theoretical perspectives introduced earlier: use-dependent plasticity (Zatorre et al., 2012) and the neural efficiency hypothesis (Poldrack, 2000). Together, these frameworks provide a useful lens for understanding why some studies report increases in structural or functional measures, whereas others report reductions or more focal engagement as learning progresses. Importantly, most of the studies included in this review used longitudinal or intervention-based designs. This strengthens the interpretation that the observed neural changes are related to language learning itself, compared with earlier research that relied mainly on cross-sectional comparisons (Table 3).

**Table 3:** Documented gaps and issues in the included studies.

Study (year)	Issue type	Description
Liu et al. (2021)	Missing control group	Lacks a non-language learning control group
Stein et al. (2012)	Small sample size/no control	*n* = 10; no control group; mixed prior L2 knowledge.
Legault et al. (2019a, 2019b)	Control group protocol	Control group data from a previous study with a slightly different scanning protocol.
Rossi et al. (2017)	Correlation vs. causation	Cross-sectional; cannot determine if WM differences are cause or consequence of bilingualism.
Bubbico et al. (2019)	Baseline group differences	Groups differed in age, education, and MMSE at baseline, though statistically controlled.
Nilsson et al. (2021)	Null finding	Well-powered RCT found no structural changes despite behavioral learning.
Fong et al. (2022)	Missing Post-MRI/control	No post-learning MRI to measure change; no control group.
Bubbico et al. (2025)	Small sample/protocol	Pilot study with small sample (*n* = 27); slightly different fMRI protocols between groups.
Schultz et al. (2024)	Missing control group	Pre-post design without a control group, limiting causal inference.

This table catalogues the common methodological challenges and reporting issues encountered in the included studies, such as small sample sizes and limited control groups, which are crucial for interpreting the current state of the field and guiding future research.

It is also important to interpret these findings within the broader literature on experience-dependent neuroplasticity. Some of the structural and functional changes reported during adult L2 learning may reflect mechanisms that are not unique to language, but are shared with other complex forms of learning, including motor skill acquisition, musical training, navigation learning, and cognitive training. From this perspective, increases in cortical thickness, gray matter volume, white matter integrity, or functional connectivity may partly reflect domain-general adaptation to sustained cognitive demand, attentional control, memory load, and repeated skill practice. The language-specific contribution may lie in the particular combination of lexical-semantic learning, phonological processing, grammatical integration, and language-control demands imposed by L2 learning.

This review was conducted as a systematic scoping synthesis, rather than a meta-analysis-driven systematic review. The neuroimaging literature on adult L2 learning is characterized by pronounced heterogeneity in study design, outcome metrics, analytical pipelines, and participant profiles, precluding meaningful quantitative aggregation. A scoping approach therefore allows for rigorous mapping of evidence, identification of boundary conditions, and critical evaluation of methodological strengths and limitations, while avoiding spurious quantitative inference. Accordingly, this review emphasizes evidence mapping and critical integration rather than quantitative aggregation, consistent with best practice for heterogeneous neuroimaging literature.

The principal findings reveal that the adult brain retains a robust, albeit modulated, capacity for experience-dependent reorganization in response to L2 learning. The evidence confirms that this plasticity is not monolithic, but involves a complex interplay of expansion and refinement across the brain’s architecture. Structurally, longitudinal studies indicate that intensive L2 learning in young adults is associated with increases in gray matter within a core language network including the (IFG), superior and middle temporal gyri, anterior cingulate cortex, and hippocampus (Stein et al., 2012; Legault et al., 2019a; Fong et al., 2022). Alongside these changes also in longitudinal studies, gradual alterations are observed in white matter, such as increased FA in frontal tracts (Schlegel et al., 2012) and dynamic reorganization of intra- and inter-hemispheric connectivity (Wei et al., 2024). Functionally, another longitudinal study indicated that L2 learning is marked by the refinement and expansion of activation patterns, evidenced by increased recruitment of areas such as the SMA and angular gyrus in proficient learners (Yang et al., 2015). Critically, these alterations are not assured; age and training intensity emerge as candidate factors that may influence neuroplastic outcomes. Nilsson et al. (2021) found that, in a randomized controlled trial, structural brain changes in older adults were modest and less pronounced, but still demonstrate significant functional reorganization (Bubbico et al., 2025; Bubbico et al., 2019; Schultz et al., 2024). The collective evidence illustrates a dynamic system that enhances its resources by selective pruning and growth, contesting simplistic narratives of linear progression.

### Interpretation of structural plasticity

The identified structural changes serve as neural correlates for the cognitive requirements of L2 acquisition. The persistent increase of gray matter in frontal (IFG, MFG) and temporal (STG, MTG) regions corresponds with their recognized functions in lexical retrieval, grammatical processing, and auditory-phonological integration (Abutalebi and Green, 2016). This pattern strongly suggests use-dependent plasticity (Zatorre et al., 2012). The involvement of the hippocampus reported in several L2 learning studies highlights the contribution of declarative memory systems to the acquisition and consolidation of new lexical and grammatical knowledge.

However, this interpretation is complicated by longitudinal observational reports of gray matter decreases in regions like the left ACC and right IFG, which correlated with improved language switching ability (Liu et al., 2021). This pattern corresponds with the brain efficiency hypothesis, which posits a transition from an initial state of effortful processing to more automated and streamlined activity. Synaptic pruning—the removal of redundant connections to improve processing efficiency—may explain such changes (Poldrack, 2000). These variations may reflect strategic optimization as part of the brain’s structural response, which may differ according to the learner’s proficiency level and the specific cognitive demands of the learning context, including language exposure intensity, linguistic complexity, memory requirements, and executive-control demands associated with language use and management.

In white matter, the progressive increases in FA found in longitudinal cohort studies (Schlegel et al., 2012; Wei et al., 2024) suggest improved myelination and axonal integrity in tracts that facilitate rapid communication within the language network. The large-scale reorganization reported by Wei et al. (2024)—increased intra-hemispheric connectivity in conjunction with decreased inter-hemispheric connectivity via the corpus callosum—hints at a fundamental rewiring of the brain’s communication highways to more efficiently support lateralized language functions. Structural expansions observed in frontal and temporal language regions following intensive training are consistent with use-dependent plasticity, whereby sustained cognitive demand leads to increased recruitment and remodeling of neural resources. From this perspective, early or intensive stages of L2 learning may be characterized by transient expansion of cortical and white matter structures that support lexical acquisition, phonological processing, and cognitive control. This finding moves beyond the simple idea that “more is better,” indicating a nuanced reconfiguration of information flow to facilitate the development of new linguistic proficiency. However, structural alterations should be interpreted in conjunction with functional findings, as changes in brain architecture do not necessarily translate directly into altered neural dynamics.

### Functional reorganization and network dynamics

Functional reorganization observed during or after language learning may occur in the absence of detectable structural change, reflecting adaptive reweighting within existing neural circuits. Functional neuroimaging reveals dynamic alterations in cerebral activity that support behavioral improvements. The shift to proficient L2 processing is characterized by an enhancement of neuronal responses. Successful learners have a more coherent and extended neural network for demanding tasks such as tone discrimination, ultimately engaging the SMA and angular gyrus in longitudinal control study (Yang et al., 2015). This indicates that proficiency involves not only activation of the appropriate areas, but also coordination of a more efficient and collaborative network. Resting-state fMRI evidence indicates that L2 learning in older adults can enhance intrinsic connectivity within prefrontal and language-control networks, suggesting that training-related plasticity may also manifest in the brain’s intrinsic functional architecture (Bubbico et al., 2025; Bubbico et al., 2019).

An important consideration indicated by the reviewed literature is the relationship between structural plasticity and functional reorganization. Although these domains are often examined separately, they likely reflect complementary aspects of experience-dependent brain adaptation. Functional reorganization may occur relatively rapidly in response to learning demands, with detectable structural changes emerging more gradually, or only under sustained and intensive training. Moreover, existing structural architecture may constrain the range of functional configurations that can be expressed, leading to variability in structure–function coupling across studies and neural systems.

### Age- and intensity-dependent modulation

A critical finding of this review is the large disparity in neuroplastic outcomes between younger and older adults. In young adults, intensive training regimens and immersive learning contexts consistently elicit structural changes within language-related cortical regions (Stein et al., 2012; Legault et al., 2019b). The current evidence base for structural plasticity in older adults remains sparse relative to younger cohorts; this pattern may reflect the underrepresentation of older individuals in longitudinal and intervention-based neuroimaging research, rather than an absence of plastic change. Despite the effective acquisition of vocabulary, the well-powered RCT conducted by Nilsson et al. (2021) did not detect any significant changes in gray or white matter in older adults following an 11-week language course. This implies that the decoupling of behavioral gains from macroscopic brain change may be due to age-related constraints on morphological plasticity.

This does not mean, however, that the older brain is impervious to change. Functional plasticity appears to be more persistent. Bubbico et al. (2025), Bubbico et al. (2019), and Schultz et al. (2024) found that L2 learning in older individuals modulates task-based activation and improves functional connectivity, enhancing global cognition and executive function. The aging brain may be less dependent on structural remodeling to achieve learning, and more so on functional rearrangement and network-level compensation, according to this dissociation. Importantly, training intensity is a critical boundary condition; the functional system remains responsive, but the shorter, less intensive courses frequently employed with older individuals may not be sufficient to induce structural change.

### Integration across modalities

When integrated, the multi-modal evidence aligns closely with existing theoretical frameworks, particularly the Dynamic Restructuring Model of bilingual neuroplasticity proposed by Pliatsikas (2020). This model posits an initial phase of neural expansion followed by refinement and efficiency optimization. The present review does not propose a novel theoretical model, but rather evaluates the extent to which longitudinal and multimodal neuroimaging evidence in adult L2 learners supports, refines, or constrains this framework across different ages, training intensities, and study designs.

Consistent with this Dynamic Restructuring Model (Pliatsikas, 2020), early stages of learning may be associated with use-dependent structural expansion, followed by a transition toward neural efficiency as proficiency increases. This is further enhanced by the strategic rewiring of white matter pathways to optimize information transfer. The functional changes in both task-based activation and resting-state connectivity presumably represent the dynamic expression of this evolving structural framework. For example, longitudinal evidence of increased fractional anisotropy in frontal tracts during L2 learning (Schlegel et al., 2012) may reflect improved efficiency of information transfer within language and cognitive control networks that support increasing linguistic proficiency.

### Comparisons with existing literature

Our findings largely corroborate and extend the frameworks established by prior neuroplasticity and bilingualism research. The involvement of the left IFG, STG, and associated control regions aligns with well-established models of language processing and cognitive control in multilingual language use (Abutalebi and Green, 2016). Cross-sectional variations in white matter organization alone should not be interpreted as evidence of learning-related plasticity, especially when such differences may reflect pre-existing neural predispositions rather than learning-induced change. Accordingly, more robust conclusions regarding experience-dependent white matter change are typically derived from longitudinal or intervention-based research designs. However, by emphasizing longitudinal evidence of learning, this review enhances the strength of inferences concerning learning-related neural change, while mitigating—though not fully eliminating—the influence of pre-existing individual differences. These findings offer more compelling support for learning-related neuroplastic processes. Nevertheless, firm causal interpretation remains limited by methodological design and contextual considerations.

The observed increases and declines in brain volume match Pliatsikas’s (Pliatsikas, 2020) dynamic model of the bilingual brain, which predicts initial expansion followed by specialization and efficiency. Conversely, our findings challenge an oversimplified application of the cognitive reserve model (Stern, 2009). The longitudinal intervention by Nilsson et al. (2021) in older adults did not produce similar outcomes. This suggests that the correlation between bilingualism, L2 acquisition, cerebral architecture, and cognitive reserve is non-linear and probably affected by variables such as age of acquisition, proficiency, and the characteristics of language utilization throughout life.

### Implications for cognitive reserve and lifelong learning

The potential of L2 learning to contribute to cognitive reserve in aging is one of its most compelling societal implications. The reported functional and connectivity improvements in older learners (Bubbico et al., 2025; Bubbico et al., 2019; Schultz et al., 2024) are encouraging. These findings suggest that, despite the lack of significant structural alterations, active engagement in second-language learning during later adulthood, including the acquisition of a new language and the further development of existing L2 skills through structured learning interventions—can elicit functional neuroplasticity associated with improved cognitive performance and may contribute to resilience against age-related cognitive decline. This functional reorganization may be a critical method for older individuals to address neural issues by more effectively utilizing brain networks.

However, the current evidence should temper excessive optimism. The absence of structural plasticity in some intervention studies suggests that potential cognitive benefits may be more closely related to ongoing cognitive engagement during learning rather than the accumulation of a permanent structural reserve. This suggests that ongoing, rigorous involvement may be needed to retain functional benefits, establishing L2 learning as a lifelong mental exercise rather than a one-time cognitive protection.

Conceptually, this review should be understood as theory-guided evidence mapping exercise, rather than the introduction of a new neuroplasticity model.

### Methodological considerations and limitations

The synthesis of this literature is limited by several methodological problems. A significant limitation across many included studies was their small sample size, which reduces statistical power and generalizability. The frequent lack of an active control group in longitudinal designs (Liu et al., 2021; Fong et al., 2022) makes it difficult to eliminate confounding variables, such as general cognitive stimulation or test–retest effects. Moreover, significant variability exists in L2 learning modes (classroom, immersion, digital), imaging modalities, and analytical frameworks, complicating direct comparisons across studies.

The fundamental issue of whether observed neural differences are the cause or consequence of language proficiency remains difficult to resolve in studies that rely primarily on cross-sectional comparisons rather than longitudinal learning design (Rossi et al., 2017). In addition, bias toward specific methodologies exists in the field, with sMRI studies being the most prevalent. A more comprehensive understanding of learning-related neuroplasticity will require multimodal designs integrating structural MRI, diffusion imaging, and functional MRI across multiple stages of language acquisition. Finally, in line with the scope defined at the time of protocol registration, this review focused on MRI- and EEG-based neuroimaging studies, and did not include work using functional near-infrared spectroscopy (fNIRS). As fNIRS is increasingly used to study language learning and brain dynamics, especially in settings where MRI is less feasible, future reviews may benefit from incorporating this growing body of evidence.

Overall, the strength of inferences drawn from this literature is constrained by small sample sizes, methodological heterogeneity, and the limited representation of older adults and well-controlled intervention studies.

### Future directions

Next-generation research should prioritize several critical areas to advance this field. First, there is a pressing need for longitudinal, multi-modal studies that track the same individuals using sMRI, DTI, fNIRS, fMRI, and EEG, from a monolingual baseline through various stages of proficiency. This would provide a direct examination of the temporal dynamics and causal relationships between structural and functional alterations. Secondly, lifetime comparisons are essential to define age limitations by comparing the neuroplastic trajectories of young, middle-aged, and older people in identical learning paradigms. Third, future experiments should ensure the use of active control groups, larger samples, and pre-registered hypotheses to improve causal inference. Fourth, exploring the ecological validity of digital and informal learning platforms, and their capacity to induce neuroplasticity, will be crucial for translating these findings into scalable cognitive health interventions for the general public. These issues should be addressed so that future research can go beyond simply describing brain changes, to become a predictive science of how, when, and for whom L2 learning impacts the mind and brain.

Finally, future research would also benefit from directly comparing the neural adaptations associated with second-language learning to those observed in other forms of complex skill acquisition, including musical training, motor learning, navigation, and cognitive training. Such comparisons may help distinguish language-specific neuroplastic mechanisms from domain-general processes of experience-dependent brain reorganization. Understanding the extent to which structural and functional changes are observed reflect unique linguistic demands versus broader learning-related adaptations remains an important challenge for future neuroimaging research.

## Conclusion

In conclusion, this systematic scoping review indicates that adult L2 learning induces significant, multifaceted neuroplasticity. The adult brain dynamically reconstitutes itself, demonstrating structural expansion and refinement in language and control networks, alongside functional reorganization to improve cognitive efficiency. Structural and functional findings underscore that neuroplasticity in adult L2 learning is a multi-level process, with dynamic interactions between brain architecture and patterns of neural activity. Although these plastic changes are robust in young adults, they are influenced by age and learning intensity, with older learners exhibiting more pronounced functional adaptation than structural adaptation. While these findings suggest that adult L2 learning is associated with detectable neuroimaging changes, the magnitude, durability, and moderators of these effects remain incompletely characterized. Ultimately, adult L2 learning provides a valuable model of experience-dependent neuroplasticity, while also requiring careful distinction between explicit learning, immersion-based acquisition-like processes, and domain-general mechanisms of skill-related brain adaptation.

Although functional brain reorganization can be observed in older learners, claims regarding structural plasticity and cognitive reserve in aging should be regarded as preliminary and contingent on future longitudinal and interventional evidence.

## Data Availability

The original contributions presented in the study are included in the article/supplementary material, further inquiries can be directed to the corresponding author.
